# Concomitant Pre- and Post-splenectomy Physiotherapy Rehabilitation in a 17-Year-Old Patient With Beta Thalassemia Major: A Case Report

**DOI:** 10.7759/cureus.29999

**Published:** 2022-10-06

**Authors:** Chitrakshi A Choubisa, Moli Jain, Vishnu Vardhan, Yukta J Budhwani, Akanksha R Hege

**Affiliations:** 1 Department of Cardiorespiratory Physiotherapy, Ravi Nair Physiotherapy College, Datta Meghe Institute of Medical Sciences, Wardha, IND

**Keywords:** thalassemia, beta thalassemia, beta thalassemia major, functional rehabilitation, pre and post-operative physiotherapy management, abdominal surgery, splenectomy

## Abstract

Thalassemia is a group of disorders having hematological origin. It is hereditary in nature, characterized by a defect in the synthesis of alpha or beta chains of hemoglobin leading to alpha or beta thalassemia, respectively. Based on the severity, beta thalassemia can be minor, intermittent, or major. Patients with thalassemia major require frequent blood transfusions, which come with various complications, of which hepatosplenomegaly is the most common. A 17-year-old male patient had a chief complaint of stomach ache and fever for the last five days. He was on a monthly blood transfusion. USG impression revealed hepatosplenomegaly and cholelithiasis. Splenectomy along with cholecystectomy was done as a part of surgical management after which a comprehensive pre- and postoperative physiotherapeutic rehabilitation program has been inculcated incorporating various respiratory techniques, strength training, and home exercise program, hence helping the patient to return to his routine daily activities efficiently. The Numeric Pain Rating Scale, Fatigue Severity Scale, and Beck Anxiety Inventory were used as outcome measures over four weeks to demonstrate the efficacy of the treatment. In this case study, a well-planned comprehensive physiotherapy rehabilitation protocol has proven helpful in improving quality of life, maximizing functional capacity, and reducing anxiety and depression in the patient.

## Introduction

Beta thalassemia is a hematological disorder in which there is reduced or absent production of the beta globulin chain affecting about 5% of people around the globe [1,2]. Every year, approximately 70,000 children are born with different kinds of thalassemia, of which 50% have beta thalassemia [2]. Regular lifelong red blood cell transfusions [3] and iron chelator therapies are the mainstays of beta thalassemia major treatment [4] with prevention of iron overload-related complications such as bone marrow hypertrophy with bony deformities and osteoporosis, hepatosplenomegaly, cholelithiasis, and lower heart rate recovery causing cardiac complications [5]. The defective proliferation of red blood cells results in their destruction in the spleen resulting in splenomegaly and the subsequent need for recurrent blood transfusions. Splenectomy plays a pivotal role in managing anemia; however, it is associated with its own set of complications including increased risk of infections, hypercoagulability, and thromboembolism [3]. Owing to this reason, splenectomy, a very impactful surgical procedure, can be considered a key part of treatment and thus decreases blood exhaustion [6,7]. Apart from anatomical and physiological changes associated with thalassemia, there is an increased rate of various psychological problems such as depression and anxiety [2]. Improved transfusion programs, iron chelation therapy, efficient treatment of complications, comprehensive care, and early physiotherapeutic rehabilitation with 30-minute preoperative sessions including inspiratory muscle training significantly improve respiratory muscle function in the early postoperative period, thereby enabling children with thalassemia to live a relatively normal life with improved quality of life [8]. Various techniques of breathing along with positive expiratory pressure are likely to have a beneficiary effect on respiratory muscle strengthening in patients undergoing abdominal surgery [9,10].

## Case presentation

Patient information

**Figure 1:**
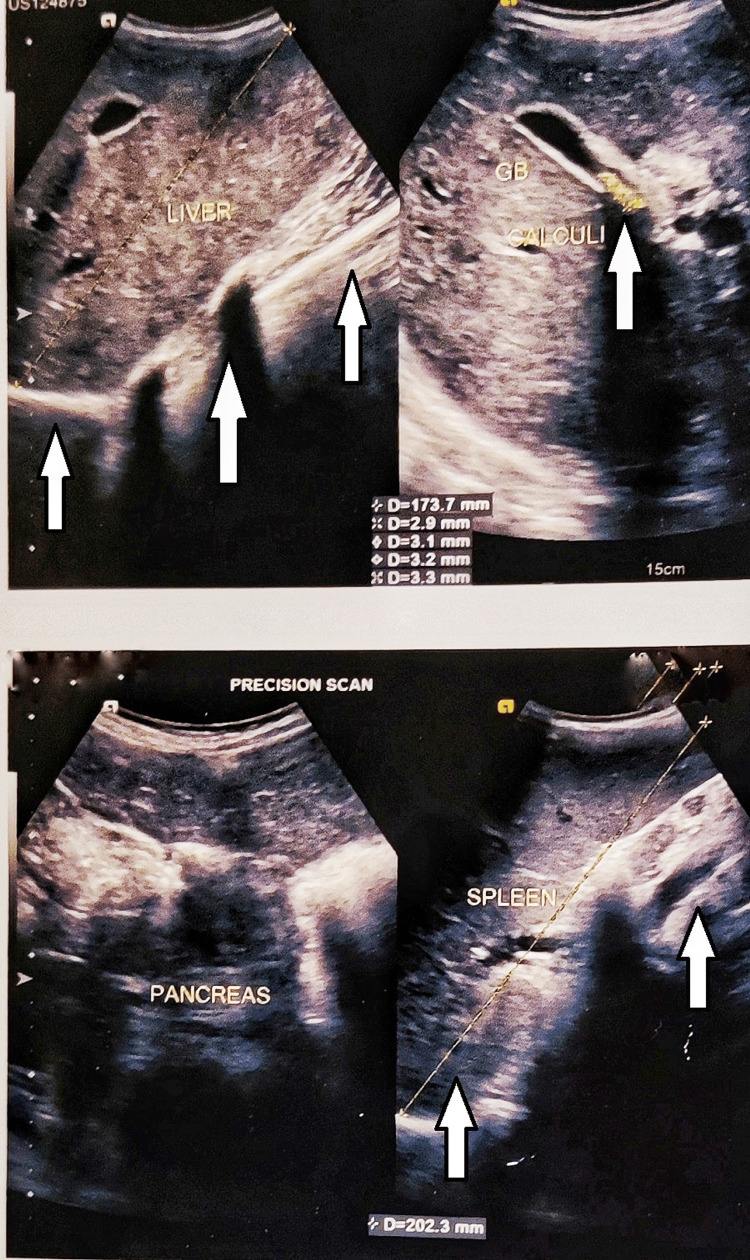
Ultrasonography of the abdomen reveals enlarged liver with a size of 17.3 cm, an enlarged spleen with a size of 20.2 cm, and multiple calculi at the neck of the gall bladder measuring 6 × 6 mm in size

A 17-year-old male patient, a known case of beta thalassemia major, reported to a rural hospital on May 5, 2022, with the chief complaint of stomach ache and fever for five days. The pain was acute in onset and gradually progressing, and present over the left upper quadrant. The patient was on a monthly blood transfusion; the last date of the blood transfusion was April 13, 2022. Following evaluation, he was admitted for further workup. Ultrasonography revealed hepatosplenomegaly and cholelithiasis for which he was advised operative management (Figure 1). He was vaccinated for preventing pneumococcal and meningococcal infections. On May 15, 2022, he underwent splenectomy along with cholecystectomy.

Clinical findings

**Figure 2:**
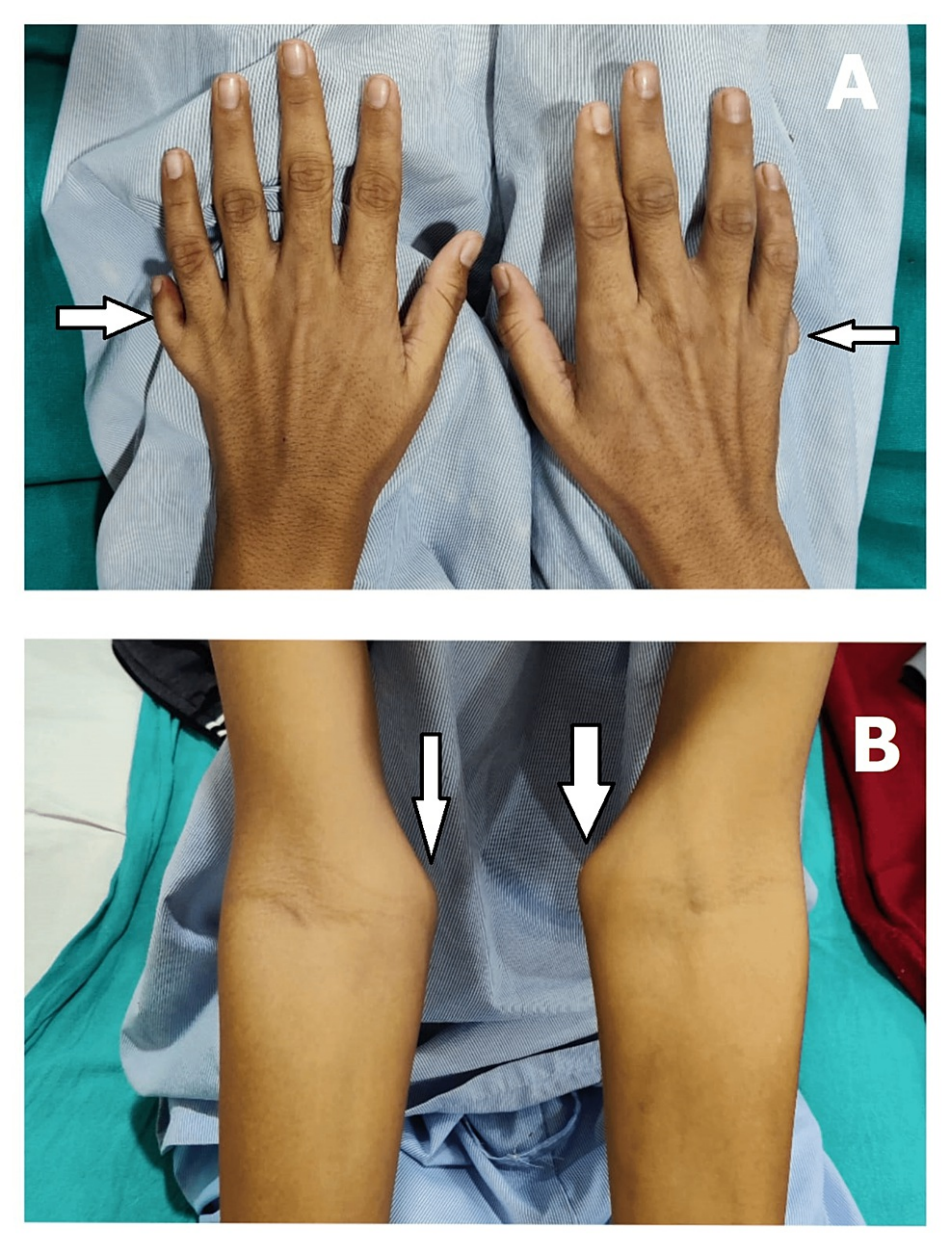
(A) Polydactyly and (B) widening of bony prominences of long bones

Informed consent was taken from the patient’s caregiver prior to the examination and the patient was positioned in a supine lying position. The patient was conscious, oriented, and obeyed commands. The patient was ectomorphic, with a BMI of 16.6 kg/m2, which is considered underweight. Preoperatively, the patient was experiencing pain in the left lower quadrant, which was insidious in onset and gradually progressive in nature. The patient rated the pain 9/10 on the Numeric Pain Rating Scale. Ryle's tube, Foley catheter, and central line were in situ. Widening of bony prominences of long bones (Figure 2A) and polydactyly was seen on all four limbs (Figure 2B). The patient was experiencing pain at the site of the suture, which was sudden in onset and dull aching in nature, which he rated 6/10 on the Numeric Pain Rating Scale. The pain aggravated while coughing and turning in bed and he rated it 8/10. The pain was relieved with rest and medication. Cardiovascular and respiratory examination revealed normal precordium, which was bilaterally symmetrical, apex impulse was present over the fifth intercostal space, and the suture was present on the upper abdominal region. The pulse rate was 76 beats per minute, rhythmic and regular, and all peripheral pulsations were present. Respiratory rate was 18 breaths per minute, with reduced chest expansion in the middle and lower zones.

Timeline

The timeline has been illustrated in Table 1 along with a description of events since admission to the hospital.

**Table 1:** Timeline

S. No.	Date of events	Consultation	Description of events
1.	5^th^ May 2022	Admission to hospital	Stomach ache and fever
2.	6^th^ May 2022	Preoperative physiotherapy	Education of patient about the condition and physiotherapy intervention
3.	15^th^ May 2022	Surgery	Splenectomy along with cholecystectomy using chevron incision
4.	16^th^ May 2022	Postoperative physiotherapy	Deep breathing exercises, spirometry, and splinted coughing
5.	3^rd^ June 2022	Discharge	Independent activities of daily living, strengthening exercise, and the patient discharged with a proper home exercise program
6.	9^th^ June 2022	Follow up	Strengthening exercises were continued. Advised patient to be as active as possible

Diagnostic assessments

The diagnostic assessments of the patient are illustrated in Table 2.

**Table 2:** Diagnostic outcomes INR: international normalized ratio; LFT: liver function test; ALT: alanine transaminase; AST: aspartate aminotransferase; CBC: complete blood count; RBC: red blood cells; Hb: hemoglobin; ABG: arterial blood gas; PCO2: partial pressure of carbon dioxide; PO2: partial pressure of oxygen; HCO3: bicarbonate.

S. No.	Investigation	Preoperative findings	Postoperative findings
1.	Ultrasonography abdomen	Enlarged liver with a size of 17.3 cm. Enlarged spleen with a size of 20.2 cm. Multiple calculi at the neck of the gallbladder measuring 6 × 6 mm in size	Nil
2.	Prothrombin time	15.7 seconds	13.2 seconds
3.	INR	1.33	1.1
4.	LFT	Total bilirubin: 4.8 mg/dl; total protein: 10.7; albumin: 4.9; alkaline phosphatase: 153; ALT: 80; AST: 86	Total bilirubin: 2.5 mg/dl; total protein: 5.6; albumin: 3.0; alkaline phosphatase: 78; ALT: 67; AST: 73
5.	CBC	Total RBC: 3.97 cells/mcL; Hb %: 7.2	Total RBC: 4.48 cells/mcL; Hb %: 9.6
6.	Serum ferritin	1604 ng/ml	270 ng/dl
6.	ABG	Ph: 7.510; PCO2: 38.8 mmhg; PO2: 51.6 mmhg; HCO3: 30.7 mEq/L	Ph: 7.423; PCO2: 37.5 mmhg; PO2: 80 mmhg; HCO3: 28.7 mmhg
7.	Peripheral smear	RBCs seem microcytic hypochromic with anisopoikilocytosis showing few pencil cells, occasional teardrop cells, and fragmented RBCs have been seen	RBCs seem to be predominantly normocytic, mildly hypochromic with mild anisopoikilocytosis showing few microcytes, occasional pencil cells, and few nucleated RBCs have been seen

Therapeutic intervention

**Figure 3:**
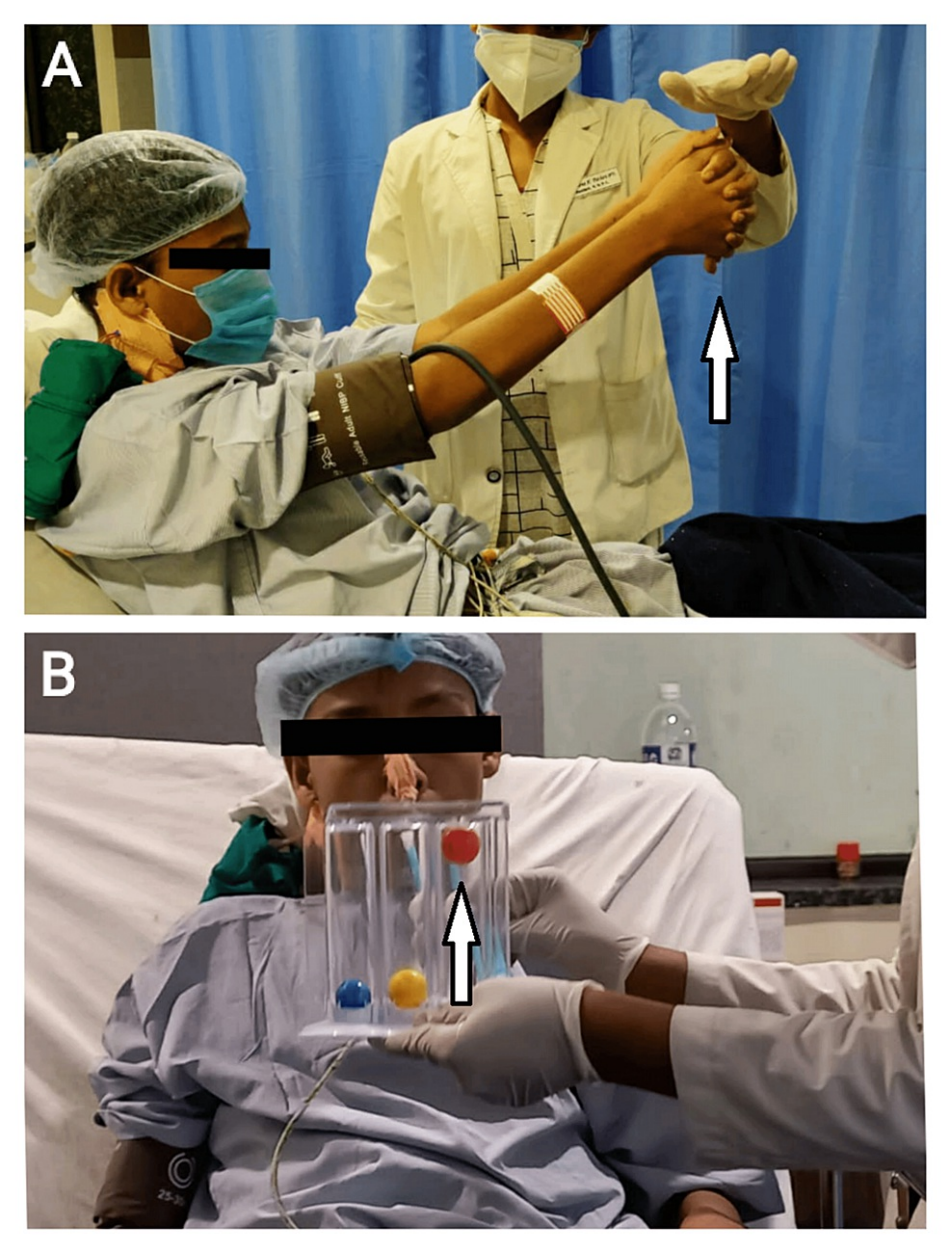
(A) Postoperatively in surgery ICU, the patient is performing thoracic expansion exercise. (B) The patient using an incentive spirometer

**Figure 4:**
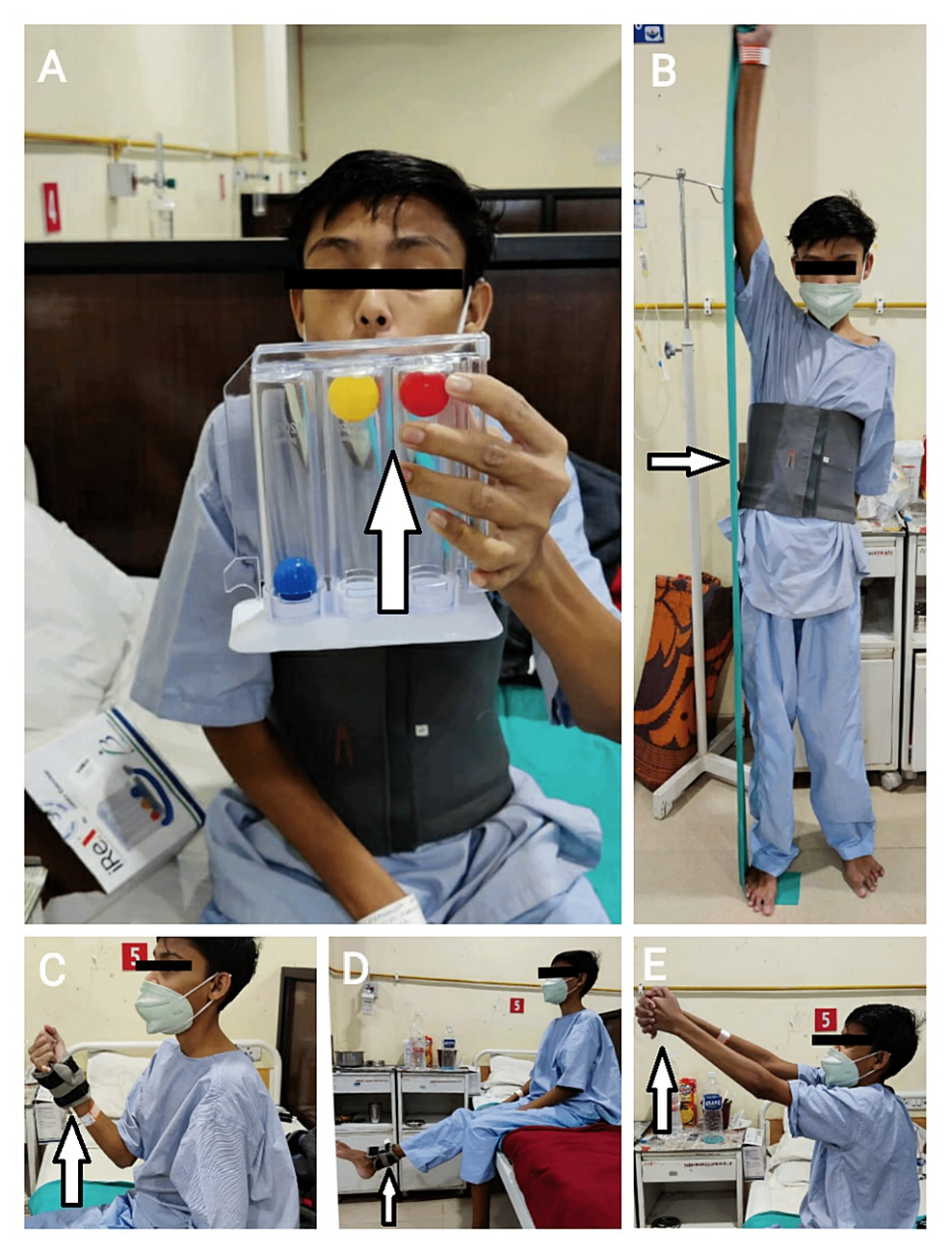
(A) Postoperatively in the surgery ward, the patient is performing incentive spirometer exercise, strength training of upper and lower limbs with theraband (B) and 1 kg weight cuff (C and D), and range of motion exercise (E)

Physiotherapy management in pre- and postoperative phases is illustrated in Table 3 and Figures 3, 4.

**Table 3:** Physiotherapy interventions

Goals	Physiotherapy intervention	Rationale
Preoperative interventions:		
To educate the patient about the condition, benefits of physiotherapy intervention, and demonstration of breathing exercises and forced expiratory technique	Education and counseling of the patient and his family. Diaphragmatic breathing and huffing technique	Helps the patient to better understand the condition and perform the techniques postoperatively with ease
Postoperative intervention:		
To educate the patient about physiotherapy protocol and its benefits	Education and counseling of the patient and his family	To encourage active participation of the patient, hence increasing the efficacy of treatment
To improve respiratory function	Diaphragmatic breathing and thoracic expansion exercise (5 reps, 4-5 times a day) (Figure 3A)	Preventing adverse effects of general anesthesia and atelectasis
To maintain bronchial hygiene	Nebulization with Budecort and huffing technique with an abdominal binder for 10 minutes, 2 times a day.	Helps in maintaining the patency of airways hence improving the vital capacity
To improve the strength of respiratory muscles	Spirometer with 3-5 seconds hold and increasing up to 10 seconds hold (5 reps, 3 times a day) (Figures 3B, 4A)	Maintaining proper expansion of lungs and avoiding the use of accessory muscles
To improve and maintain the strength of upper and lower extremity muscles	1 kg weight cuffs and theraband usage (10 reps x 1 set) (Figures 4B-4E)	Reduces fatigue and improves exercise outcomes
To reduce anxiety and depression	Buteyko breathing technique: after a relaxed exhale, ask the patient to hold the breath with the index finger and thumb to plug the nose, retain the breath until the patient feels the urge to breathe, and then exhale followed by normal breathing	Helps to relax the patient
To improve quality of life	Home exercise program including deep breathing exercises, upper and lower extremities mobility and strengthening exercises, hall ambulation, and stair climbing	To maintain the gained progress and promote further improvement

Follow-up and outcomes

Preoperative and postoperative comprehensive and well-planned physiotherapy protocol along with a home exercise program was incorporated. Follow-up was done once a week for four weeks using various outcome measures illustrated in Table 4.

**Table 4:** Outcome measures

Scales	Preoperative	Postoperative day 1	Postoperative week 2	Postoperative week 4
Numeric Pain Rating Scale	8/10	8/10	6/10	5/10
Fatigue Severity Scale	5.1	5.6	4.7	3.7
Beck Anxiety Inventory	22	23	16	9

## Discussion

Beta thalassemia major is a severe type of thalassemia requiring frequent blood transfusion, which eventually leads to various inevitable anatomical and psychological complications such as osteoporosis, hepatosplenomegaly, cholelithiasis, cardiac dysfunction, anxiety, and depression [1,2,5]. Various pharmacological and surgical treatments including iron-chelating drugs, splenectomy, and cholecystectomy have shown better results in managing these complications [3,6].

According to Larsen et al., a precise and well-planned preoperative physiotherapy session has shown significant improvement in respiratory muscle function and hence halving the risk of pulmonary complications, specifically hospital-acquired pneumonia [8]. Studies done by Orman and Westerdahl showed various techniques of breathing along with positive expiratory pressure are likely to have a beneficiary effect on respiratory muscle strengthening in patients undergoing major abdominal surgery [10]. Physiotherapy management has improved the functional activity and quality of life of the patient.

## Conclusions

Splenectomy following thalassemia major may have adverse effects on various other systems of the body including musculoskeletal and cardiac systems. A well-planned comprehensive physiotherapy rehabilitation protocol has proven helpful in improving quality of life, maximizing functional capacity, and reducing anxiety and depression in the patient.
